# Cancer Risk in Patients with Acromegaly: Insights from a Single Center in Ankara

**DOI:** 10.3390/jcm15041573

**Published:** 2026-02-17

**Authors:** Murat Cinel, Ozgur Demir, Rovsan Hasenov, Sule Canlar, Caglar Keskin, Asena Gökçay Canpolat, Mustafa Sahin, Sevim Güllü, Demet Corapcioglu

**Affiliations:** Endocrinology Department, Ankara University Faculty of Medicine, 06000 Ankara, Turkey

**Keywords:** acromegaly, growth hormone, insulin-like growth factor 1, cancer

## Abstract

**Background**: Acromegaly is a rare, chronic, systemic, and progressive disease characterized by an excess secretion of growth hormone (GH) and increased circulating insulin-like growth factor 1 (IGF-1) concentrations, typically due to a macroadenoma in the pituitary gland. Both GH and IGF-1 are implicated in cancer promotion based on experimental and epidemiological data, but research findings remain conflicting and population-based data are scarce. Although there is a high mortality rate among acromegalic patients due to cardiovascular diseases, cancer is the third leading cause of death. **Aim**: The aim of the present study was to assess the risk of different types of cancer in acromegaly and the impact of changes in disease control and patient outcomes over time. **Methods**: Patients diagnosed with acromegaly at the Ankara University Ibn-i Sina Hospital Endocrinology and Metabolic Diseases Department between 2015 and 2019 were included in this study. Data including demographic data, history of cancer, size of adenoma (micro or macro), serum IGF-1 and GH levels at the time of diagnosis, serum prostate-specific antigen (PSA), thyroid ultrasonography, and, if needed, thyroid fine needle aspiration cytology (TFAC), colonoscopy, and mammography results were collected from patient records retrospectively. **Results**: We screened 83 patients, and 78 patients with the compensatory data (female/male: 39/39, 50%/50%) were included. The mean age of patients was 49.4 ± 11.9 years and 41.7 ± 12.1 years at the time of diagnosis. The median duration of follow-up was 72 (12–420) months. Periodic thyroid ultrasonography was performed in 65/78 (83.3%) of the patients, and a colonoscopy and mammography were also conducted in 27/78 (34.6%) and 32/39 (82%) of the patients at least once over the course of the disease, respectively. Cancer was detected in 17/78 (21.7%) of the patients; 11/78 (14.1%) of them had well-differentiated thyroid cancer and 2/39 (5.1%) had breast cancer. Prostate cancer, renal cell carcinoma, pancreatic cancer, malignant chordoma, schwannoma, and colon cancer were detected in one patient each. The increased cancer risk in acromegalic patients did not correlate with age, sex, age at diagnosis, time to diagnosing acromegaly, duration of acromegaly, GH and IGF-1 levels at diagnosis, pituitary adenoma size, or Ki-67 levels. **Conclusions**: Cancer was detected in 21.7% of the acromegaly patients, 14.1% of whom had well-differentiated thyroid cancer. In this study, we demonstrated that thyroid cancer is the most common malignancy in Turkish acromegalic patients, consistent with the results of previous studies. The increased cancer risk in acromegalic patients did not correlate with age, sex, age at diagnosis, time to diagnosing acromegaly, duration of acromegaly, or GH and IGF-1 levels at diagnosis.

## 1. Introduction

Acromegaly is a long-standing systemic disorder that results from persistently elevated growth hormone (GH) and insulin-like growth factor-1 (IGF-1) concentrations, most often originating from a pituitary macroadenoma. During its progression, the tumor’s mass effect may produce symptoms such as headache and visual disturbances, including blurred vision or visual field defects like scotomas, while characteristic phenotypic changes—such as coarse facial features, mandibular prognathism, and enlargement of the extremities—gradually develop. This condition shortens life expectancy primarily through atherosclerotic complications, which are commonly linked to GH- and IGF-1-induced diabetes mellitus and hypertension [1].

Whether acromegaly itself contributes to cancer development and thereby to the reduced survival observed in affected patients remains uncertain. A number of investigations suggest that the growth hormone–insulin-like growth factor (GH–IGF) axis and its receptor network may be key drivers of tumorigenesis. Elevated circulating IGF-1 concentrations have been linked to an increased likelihood of various malignant transformations. Evidence connecting acromegaly and cancer, however, is inconsistent, in part because of heterogeneity in research methodologies, including differences between case–control and population-based designs. Furthermore, many contemporary studies still lack adequate statistical power to confirm the degree of cancer risk anticipated from community-based and case–control cohorts of individuals with acromegaly. Some reports indicate that acromegaly carries a heightened risk for certain malignancies—such as differentiated thyroid carcinoma, colorectal carcinoma, female breast cancer, and male prostate cancer—as well as for overall cancer incidence. Yet other publications contradict these associations. Thus, although overall mortality in acromegaly is elevated, its precise relationship with cancer remains to be clearly established [1,2,3,4,5,6].

Examining the risk of various malignancies in individuals with acromegaly, along with the impact of evolving disease control and long-term patient outcomes, is of critical importance. The results of this research will provide valuable insights into both the clinical management of acromegaly and its possible link to cancer.

## 2. Material and Methods

### 2.1. Study Protocol

The medical records of 83 individuals diagnosed with acromegaly who were evaluated at the Endocrinology and Metabolic Diseases Department of Ankara University Medical School Hospital between 2015 and 2019 were reviewed. Of these, 78 patients (39 women and 39 men; 50%/50%) fulfilled the study’s inclusion criteria and were analyzed. The criteria were as follows: (1) age at diagnosis greater than 18 years; (2) confirmed diagnosis of acromegaly for at least 12 months; (3) the acromegaly diagnosis must include

- The presence of typical clinical manifestations of the disease;
- Abnormal nadir GH levels (>1 ng/mL) during a 75 g oral glucose tolerance test (OGTT) and/or IGF-1 levels above the 90th percentile for age and sex;
- Radiological confirmation of a pituitary tumor on magnetic resonance imaging; and (4) no prior history of malignancy.

Data collected retrospectively included demographic characteristics, time elapsed since acromegaly diagnosis, baseline serum GH and IGF-1 concentrations, tumor size on pituitary MRI, treatment modalities, and remission status. Remission and disease control were determined according to the 14th consensus criteria [7]. Pathology preparations were examined immunohistochemically for hormone positivity and Ki-67 proliferation indices were noted.

Information was collected on patients who had undergone colonoscopy, mammography, breast ultrasonography, prostate ultrasonography, prostate-specific antigen (PSA) testing, and thyroid ultrasonography, either as part of routine screening or in response to clinical symptoms. Thyroid nodules measuring ≥1 cm or smaller nodules exhibiting suspicious ultrasonographic features—such as microcalcifications, marked hypoechogenicity, intranodular arterial vascularity, or irregular borders—were assessed using fine-needle aspiration biopsy.

The study protocol received approval from the Institutional Ethical Committee of Ankara University (07-294-16/11 April 2016).

### 2.2. Study Assays

Serum GH levels were quantified using a chemiluminescence assay (DPC; Immulite, Los Angeles, CA, USA) with a detection limit as low as 0.01 µg/L. Serum IGF-1 concentrations were assessed by an immunoradiometric assay (IRMA) (Immunotech SAS; A Beckman Coulter Company, Marseille, France), which was standardized against the WHO International Reference Reagent 1988, IGF-1 87/518. The analytical sensitivity of this method was 2 ng/mL. The immunoassay employed antibodies specific to IGF-1. The currently available IGF-1 reference intervals are derived from non-representative samples of the adult Caucasian population.

All thyroid ultrasonography (USG) examinations were performed by the same physician using a 14 MHz linear probe (HV-900, Hitachi, Tokyo, Japan). The thyroid volume for each lobe was estimated with the ellipsoid formula (length × thickness × width × 0.52), and the total thyroid volume was calculated as the sum of the two lobes. Thyroid tissue was considered enlarged when the total volume exceeded 18 mL in men and 13 mL in women. Fine-needle aspiration biopsy (FNAB) of all nodules was carried out using 22–27-gauge needles. The cytology smears were prepared at room temperature and air-dried.

### 2.3. Statistical Analysis

Statistical analyses were conducted using SPSS software, version 23.0. The normality of the data was assessed using the Shapiro–Wilk test, and subsequent analyses were conducted according to the normality results. Continuous variables were compared with either a two-tailed Student’s t-test or, when appropriate, a Wilcoxon rank-sum test. Categorical variables were analyzed using the chi-square test or Fisher’s exact test. Descriptive data are presented as mean ± standard deviation (SD) and as percentages. A *p*-value < 0.05 was considered indicative of statistical significance. To assess differences between patients with acromegaly who developed cancer and those without cancer, an independent-samples *t*-test was applied.

## 3. Results

A total of 78 acromegaly patients (female/male: 39/39, 50%/50%) with a mean age of 49.4 ± 11.9 years and 41.7 ± 12.1 years at the time of diagnosis were evaluated. The median duration of follow-up was 72 (12–420) months. The most common patient complaints were acral growth (85.8%), vision problems (26.9%), headache (26.9%), sweating (16.7%), and amenorrhea among women (32.6%), as well as loss of libido among men (10%) at admission. The median GH was 6.7 ng/mL, and the IGF-1 level was 882 ng/mL. Radiologically, 10/78 (12.8%) patients had microadenoma and 56/78 (71.8%) patients had macroadenoma.

Almost all patients (77/78) were treated with trans-nasal transsphenoidal pituitary surgery, but one patient refused surgery because of social problems and was treated with primary medical therapy. In total, 29/77 (37.6%) patients were successfully treated with surgery. For 13/77 (16.9%) patients, a second surgery was indicated. For 41/78 (52.5%) patients, medical therapy was used to control hormone levels, and for 7/78 (9%) patients, radiotherapy was the third choice to control the disease.

Diabetes mellitus was reported in 20/78 (25.6%) patients, hypertension in 9/78 (11.5%) patients, and other diseases, such as coronary artery disease, hypothyroidism, and allergic disorders, were detected in 7/78 (9%) patients.

The clinical, laboratory, radiological, and pathological features, as well as co-morbidities and treatment modalities of the acromegaly patients, are shown in Table 1. Cancer was detected in 17/78 (21.7%) of the acromegaly patients. In total, 11/78 (14.1%) of them had well-differentiated thyroid cancer; 2/39 (5.1%) of female acromegaly patients had breast cancer. Prostate cancer was detected in 1/39 (2.6%) of male acromegaly patients. Malignant chordoma, pancreatic cancer, renal cell carcinoma, schwannoma, and colon cancer were detected in one patient each (1.3%). Two patients had two cancers simultaneously; one female patient had both differentiated thyroid carcinoma and breast cancer, and the other had both differentiated thyroid carcinoma and colon cancer. There was no difference concerning age, sex, age at diagnosis, time to diagnosing acromegaly, duration of acromegaly, GH and IGF-1 levels at diagnosis, adenoma size, and Ki-67 levels between patients with and without cancer. A comparison of the parameters, including age, age at diagnosis, sex, time to diagnosing acromegaly, duration of acromegaly, GH and IGF-1 levels at diagnosis, adenoma size, and Ki-67 levels between patients with and without cancer, is demonstrated in Table 2. Thyroid USG was carried out in 51/78 (65.4%) patients. Total thyroid volume was increased compared to the normal population but did not differ between those with and without thyroid cancer (*p* = 0.29). A total of 31/51 (60.7%) patients had uni- or multinodular goiter, and 20/31 (64.5%) of them had nodules which were millimetric (<1 cm) in diameter. Additionally, 25/31 (80.6%) of nodular goiter patients underwent fine-needle aspiration biopsy. Total or subtotal thyroidectomy was performed in 13/25 (52%) patients according to fine-needle aspiration cytology results, and 11/13 (84.6%) of them were reported to have well-differentiated thyroid carcinoma. In total, 10/11 (91%) of thyroid malignancies were papillary thyroid carcinoma, and 1/10 (9%) was follicular carcinoma. Among papillary thyroid carcinoma cases, 8/10 (80%) patients had the classic type, while 2/10 (20%) patients had the follicular variant and only 1 case involved papillary microcarcinoma. There was no difference concerning thyroid volume, nodularity, GH and IGF-1 levels at the diagnosis of acromegaly and the diagnosis of thyroid cancer, pituitary adenoma size, and adenoma TSH staining between acromegaly patients with or without thyroid cancer. A comparison of thyroid volume, nodularity, GH and IGF-1 levels at the diagnosis of acromegaly and the diagnosis of thyroid cancer, pituitary adenoma size, and adenoma TSH staining between acromegaly patients with or without thyroid cancer is demonstrated in Table 3. Colonoscopy was performed in 27/78 (34.6%) patients. In 18/27 (66.7%) patients, no pathology was detected; in 6/27 (22.2%) patients, polyps were detected (the most common type was adenomatous polyps); in 2/27 (7.4%) patients, diverticula were detected; and colon cancer was confirmed in 1/27 (3.7%) patient.

Mammography was used as a screening tool for breast cancer in 32/39 (% 82%) female patients. Biopsy was carried out in 3 patients, and 2/32 (6.3%) cases were diagnosed as breast cancer; pathologic subtypes included invasive ductal carcinoma.

PSA was measured as a screening tool for prostate pathology in 10/39 (25.6%) male patients, and in 1/10 (10%) of the patients who were screened, prostate carcinoma was detected.

## 4. Discussion

Studies on cancer incidence in acromegalic patients still yield conflicting results. The majority of past studies have revealed an increased risk of malignancies in acromegalic patients, but others contradict this result [4,8,9,10,11]. High GH and IGF-I levels in acromegalic patients are found to be related more to age-related cancer deaths, but the reason–result relationship is not clear yet [12,13]. Growth factors may affect malignant transformation by increasing the DNA mutation risk via several signal transduction pathways in cells. GH binds to the predimerized GH receptor (GHR) on target cells located extracellularly. Activation of GHR triggers mitogenic pathways such as JAK kinase, STAT5, MAPK/ERK, and PI3K [14,15]. Exogenous GH and the expression of large amounts of GHR have been shown to be responsible for tumourigenesis in some cancers and a more aggressive clinical course in breast, colon, prostate, and thyroid cancer. Although excess GH and cancer risk are not well defined yet, cancer screening in acromegaly is highly recommended.

In our country, cancer incidence is 254/100.000. Cancer types in men of all age groups include lung (21%), prostate (12.9%), colorectal (9.3%), and thyroid (2.7%) cancer, and cancer types in women of all age groups include breast (24.7%), thyroid (12.1%), colorectal (8.3%), and lung (5.1%) cancer [16,17]. When our study is compared with these data, the increase in thyroid cancer is remarkable.

We demonstrated that cancer incidence in our group of acromegalic patients was 21.7%. Furthermore, there was no difference between sexes. In a population-based study in our country, cancer incidence was 254.2 per 100.000 in 2018 (Turkey Cancer Control Programme), and global cancer burden in 2018, according to the WHO (GLOBOCAN research), revealed lower ratios (18.1 million new cases worldwide) [17,18]. According to these data, we assumed that cancer incidence is higher in acromegalic patients in Turkey. Cancer incidence is reported to be up to 4.8 to 21.3% among acromegalic patients, and the estimated overall standardized incidence ratio (SIR) for cancer increased up to 3.4-fold; there are also studies reporting this increase only in women [19]. In the cohort study by Jakob Dal et al., the association between acromegaly and cancer, together with the association between pituitary adenoma size, GH levels, and IGF-1 levels, supports our findings [20].

Differentiated thyroid carcinoma was the most frequently observed malignancy in our cohort, with a prevalence of 14.1%, and 91% of these thyroid cancers were of the papillary subtype. In a large contemporary series, thyroid carcinoma was identified in 6 of 331 patients (1.8%), with an equal distribution between men and women [20]. Recent large-scale investigation reported thyroid malignancy in 3 of 446 individuals (0.7%), but the small number of cases resulted in a nonsignificant standardized incidence ratio (SIR) [10]. In a meta-analysis, Wolinski et al. documented an increased overall cancer incidence rate in acromegaly with an odds ratio (OR) of 7.5, concluding that the risk of thyroid cancer is elevated in this population [18]. In another study conducted recently in Poland, it was emphasized that when benign and malignant neoplasms were evaluated together in patients with acromegaly, the most frequent lesions were located in the thyroid gland [21]. Similarly, a pooled analysis of 23 studies comprising 9677 participants demonstrated a higher risk of differentiated thyroid carcinoma (pooled SIR = 9.2; 95% CI, 4.2–19.9) [19].

Potential bias is particularly relevant for thyroid malignancies, since endocrinologists often perform thyroid ultrasonography, which can lead to overdiagnosing microscopic papillary carcinoma. In Turkey, ultrasonography is routinely employed in thyroid assessment because of its low cost, and a larger number of fine-needle aspiration biopsies may be performed in acromegalic patients given their recognized malignancy risk. Consequently, the apparent rise in thyroid cancer incidence may partly reflect a screening effect, with an increased detection of incidental micropapillary tumors. Importantly, thyroid carcinoma in patients with acromegaly typically shows a favorable prognosis and life expectancy comparable to de novo cases. In our series, one patient had micropapillary thyroid carcinoma, and no mortality was attributed to thyroid cancer; during follow-up, the disease remained in remission and under control.

Other than thyroid carcinomas, in our cancer group, patients did not show differences between thyroid nodularity (*p* = 0.27) and volume (P0:0.29), and there was no correlation with pituitary adenoma size (*p* = 0.16). There was increased nodularity (31/78 patients, % 39.7%) observed in acromegalic patients when compatible with some studies [19].

In our study group, 5.1% of female acromegaly patients suffered from breast cancer. Prostate cancer was detected in 2.6% of male acromegaly patients. Each of the malignancies, including chordoma, pancreatic cancer, renal cell carcinoma, schwannoma, and colon cancer, was detected in one patient (1.3%). Two patients had two cancers simultaneously; one female patient had both differentiated thyroid carcinoma and breast cancer, and the other had both differentiated thyroid carcinoma and colon cancer. In a meta-analysis of 23 studies, Jacob Del at al. revealed that, especially when stratified by cancer type, elevated risks were shown for colorectal cancer (pooled SIR = 2.6; increased risk of gastrointestinal malignancy (decreasing order colorectal cancer, gastric cancer)) [18]. Overall, breast and colorectal cancer incidence rates are generally high in the population. In our study, we found this ratio to be low because of the low use of gastrointestinal and mammary screening tools.

The limitations of our study are as follows: (1) the relatively low number of patients when compared with large cohorts; (2) the single-center study design; and (3) the lack of a control group to compare the risk of malignancy. The strengths of our study are as follows: (1) patients diagnosed with cancer in the last year were excluded to prevent bias; (2) although this is a single-center study, the number of patients is sufficient for a single center; and (3) there was a high incidence of differentiated thyroid malignancy.

## 5. Conclusions

In our study, cancer was detected in 21.7% of acromegaly patients, higher than the mean cancer incidence of 10.8%, with reported percentages varying from 4.8 to 21.3% in other studies. The most common malignancy in our patients was differentiated thyroid carcinoma (14.1%), which is also higher than in other series. The apparent increase in thyroid cancer incidence can be attributed to a screening effect linked to the higher use of thyroid ultrasonography and thyroid fine-needle aspiration biopsy, which has yielded a high number of ‘incidentally’ discovered less aggressive thyroid tumors. The increased cancer risk in acromegalic patients did not correlate with age, sex, age at diagnosis, time to diagnosing acromegaly, duration of acromegaly, or GH and IGF-1 levels at diagnosis.

## Figures and Tables

**Table 1:** Clinical, laboratory, radiological, and pathological features, as well as co-morbidities and treatment modalities of acromegaly patients.

Variable	Acromegaly Patients
Basics (*n* = 78)	
Age (mean ± SD) (years)	49.4 ± 11.9
Age at Diagnosis (mean ± SD) (years)	41.7 ± 12.1
Sex (female/male) (*n*, %)	39 (50%)/39 (50%)
Time to Diagnosis (median (min–max)) (months)	24 (16–70)
Duration of Disease (median (min–max)) (months)	72 (12–420)
Acromegaly-Related Complaints (*n* = 78)	
Acral Growth	67 (85.8%)
Vision Problems	21 (26.9%)
Headache	21 (26.9%)
Sweating	13 (16.7%)
Laboratory Values (at Diagnosis) (*n* = 78)	
GH (ng/mL) (median + (min–max))	6.7 (0.2–65)
IGF-1 (ng/mL) (median + (min–max))	882 (307–4000)
Pathology (*n* = 77)	
Macroadenoma (*n*, %)	56 (72.7%)
Microadenoma (*n*, %)	10 (12.9%)
Additional PRL Staining (*n*, %)	44 (57.1%)
Additional ACTH Staining (*n*, %)	9 (11.6%)
Additional TSH Staining (*n*, %)	12 (15.5%)
Additional FSH, LH Staining (*n*, %)	12 (15.5%)
Ki-67 < 3 (*n*, %)	30 (38.9%)
Ki-67 ≥ 3 (*n*, %)	21 (27.2%)
Treatment Modalities (*n* = 78)	
Surgery Alone (*n*, %)	29 (37.1%)
Surgery + Medical Therapy (*n*, %)	41 (52.5%)
Surgery + Medical + Radiotherapy (*n*, %)	7 (9%)
Primary Medical Therapy (*n*, %)	1 (1.3%)
Comorbidities (*n* = 78)	
Diabetes Mellitus (*n*, %)	20 (25.6%)
Hypertension (*n*, %)	9 (11.5%)
Other (*n*, %)	7 (9%)

GH: growth hormone; IGF-1: insulin like growth hormone-1; Ki-67: Ki-67 proliferation index.

**Table 2:** Comparison of features of acromegaly patients with and without cancer.

Variable	Acromegaly Patients with Cancer	Acromegaly Patients Without Cancer	*p* Value
Age (mean ± SD) (years), *n* = 78	49.1 ± 9.8	49.5 ± 12.5	0.920 *
Age at Acromegaly Diagnosis (mean ± SD) (years), *n* = 78	42.8 ± 10.0	41.0 ± 12.7	0.593 *
Sex (female/male) (*n*, %), *n* = 78	10 (25.6%)/7 (17.9%)	29 (74.4%)/32 (82.1%)	0.411 **
Time to Diagnosis (median + (min–max)) (months), *n* = 78	24 (6–360)	24 (2–120)	0.613 ***
Duration of Disease (median, months), *n* = 78	88 (48–408)	90 (4–312)	0.607
GH at Diagnosis of Acromegaly (ng/mL) (median + (min–max)), *n* = 78	6 (1.3–30)	6.8 (0.2–65)	0.548 ***
IGF-1 at Diagnosis of Acromegaly (ng/mL) (median + (min–max)), *n* = 78	944 (307–4000)	844 (343–3900)	0.539 ***
Adenoma (micro/macro) (*n*, %), *n* = 66	4 (23.5%)/13 (76.5%)	6 (12.2%)/43 (87.8%)	0.584 ****
Ki-67 (<3/≥3) (*n*, %), *n* = 51	3 (30%)/7 (70%)	27 (65.9%)/14 (34.1%)	0.070 ****

GH: growth hormone; IGF-1: insulin like growth hormone-1; Ki-67: Ki-67 proliferation index. * Independent-samples *t*-test; ** Pearson chi-square test; *** Mann–Whitney U test; **** Fisher’s exact test. %: Presented as column percentage.

**Table 3:** Comparison of some features of acromegaly patients with and without thyroid cancer.

Variable	Acromegaly Patients with Thyroid Cancer	Acromegaly Patients Without Thyroid Cancer	*p* Value
Thyroid Volume, *n* = 52	66 (22–107)	23 (3–67)	0.29 *
Thyroid Nodularity (uninodular/multinodular/diffuse) (*n*, %), *n* = 51	0 (0%)/3 (75%)/1 (25%)	11 (23.4%)/17 (36.2%)/19 (40.4%)	0.276 **
GH at Diagnosis of Acromegaly (ng/mL) (median + (min–max)), *n* = 78	6 (2.9–30)	6.8 (0.2–65)	0.906 *
IGF-1 at Diagnosis of Acromegaly (ng/mL) (median + (min–max)), *n* = 78	1259 (414–4000)	844 (307–4000)	0.257 *
GH at Diagnosis of Thyroid Cancer (ng/mL) (median + (min–max)), *n* = 11	1.1 (0–30)	1.4 (0–7)	1 *
IGF-1 at Diagnosis of Thyroid Cancer (ng/mL) (median + (min–max)), *n* = 11	449 (251–2017)	332.5 (65–796)	0.144 *
Pituitary Adenoma (micro/macro) (*n*, %), *n* = 66	3 (30%)/7 (70%)	7 (12.5%)/49 (87.5%)	0.169 **
TSH Staining of Pituitary Adenoma (negative/positive) (*n*, %), *n* = 78	10 (90.9%)/1 (9.1%)	56 (83.6%)/11 (16.4%)	1 ***

* Mann–Whitney U test; ** Pearson chi-square test; *** Fisher’s exact test. %: Presented as column percentage.

## Data Availability

No new data were created or analyzed in this study. Data sharing is not applicable to this article.
